# [Corrigendum] FXR1 promotes proliferation, invasion and migration of hepatocellular carcinoma *in vitro* and *in vivo*

**DOI:** 10.3892/ol.2026.15583

**Published:** 2026-04-09

**Authors:** Kun Zhao, Jie Gao, Jihua Shi, Chengcheng Shi, Chun Pang, Jie Li, Wenzhi Guo, Shuijun Zhang

Oncol Lett 25: 22, 2023; DOI: 10.3892/ol.2022.13608

Following the publication of the above article, an interested reader drew to the authors’ attention that, regarding the cell migration and invasion assay experiments shown in Fig. 3 on p. 6, a number of data panels showed overlapping sections (specifically, two pairs of panels in Fig. 3C and two further pairs in Fig. 3D), such that data which were intended to show the results of differently performed experiments had apparently been derived from the same original sources. Upon performing an independent analysis of the data in this paper in the Editorial Office, it came to light that a third pair of data panels in Fig. 3D were similarly affected. Furthermore, upon asking the authors for an explanation of the apparent anomalies in these figures, they identified an additional unintended image duplication in Fig 3. These errors inadvertently arose during the process of final assembly and integration of multiple image panels in this figure.

The authors were able to consult their original data, and realized how these errors had occurred. A revised version of Fig. 3, now showing the correct data for the ‘Invasion, shNC+/shFXR1#1-/TGF-β-’ (left-hand) and ‘Invasion, shNC+/shFXR1#2-/TGF-β+’ (second on the left) data panels in Fig. 3C, and the ‘Migration, shNC+/shFXR1#1-/TGF-β+’, ‘Migration, shNC-/shFXR1#1+/TGF-β+’ and ‘Migration, shNC-/shFXR1#2+/TGF-β-’ data panels in Fig. 3D, is shown on the next page. Note that some of the experimental conditions were also labelled incorrectly in the published version of this figure, and these have been corrected in the revised version. The authors regret that the errors occurred in the originally published version of this figure, although these did not grossly affect either the results or the conclusions reported in this article. All the authors agree with the publication of this Corrigendum, and thank the Editor of *Oncology Letters* for granting them the opportunity to publish this; furthermore. they apologize to the readership for any inconvenience caused.

## Figures and Tables

**Figure 3.**
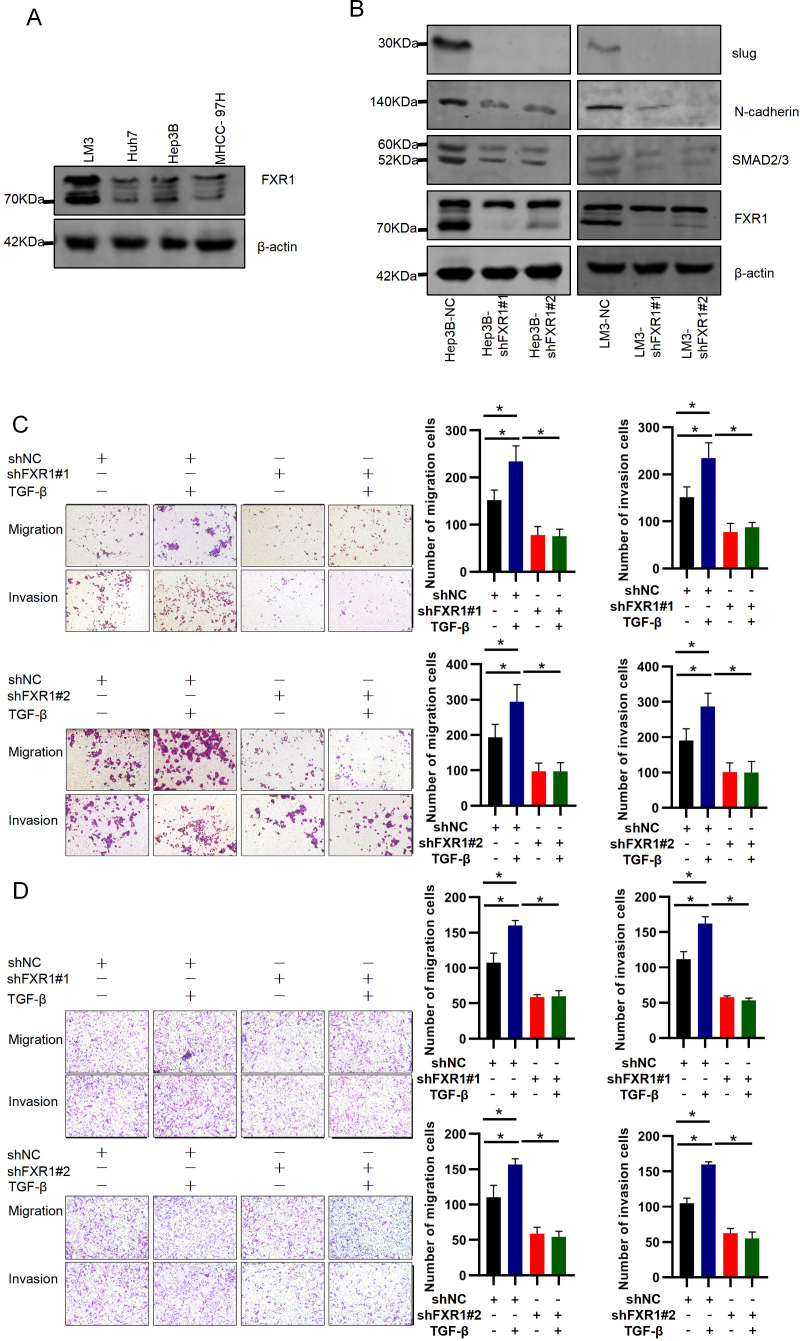
FXR1 promotes slug/N-cadherin expression and TGF-β-induced HCC cell migration and invasion. (A) FXR1 expression in HCC cells. (B) Hep3B and LM3 cells were transfected with shNC, shFXR1#1, or shFXR1#2, and western blotting was used to determine knockdown efficiency. (C) Hep3B and (D) LM3 cells were transfected with shNC, shFXR1#1, or shFXR1#2, and treated with or without TGF-β, followed by cell migration and invasion assays. The cells were then observed microscopically (magnification, ×200). The relative cell migration or invasion was plotted. Cell migration or invasion was normalized to the shNC-transfected and TGF-β-untreated group as appropriate. Note: The difference in cell status and the time of spreading the plates resulted in inconsistencies in the number of cells in the control group at different times. Data are presented as the mean ± SD of three repeats. *P<0.05. FXR1, fragile X-related 1; HCC, hepatocellular carcinoma; sh, short hairpin; NC, negative control.

